# Bacterial Toxins, Current Perspectives

**DOI:** 10.3390/toxins12090570

**Published:** 2020-09-04

**Authors:** Michel R. Popoff

**Affiliations:** Bacteries Anaerobies et Toxines, Institut Pasteur, 28 rue du Docteur Roux, 75724 Paris, France; popoff2m@gmail.com

Toxins are the major pathogenicity factors produced by numerous bacteria involved in severe diseases in humans and animals. Certain pathogenic bacteria synthesize only one toxin which is responsible for all the symptoms and outcome of the disease. For example, botulinum toxins (BoNTs) and tetanus toxin (TeNT) are the unique causal factors of botulism and tetanus, respectively. Other bacteria attack the host organism by a set of multiple toxins which synergistically act to promote the disease. This is the case of *Clostridium* and *Staphylococcus* strains which secrete wide ranges of toxins such as pore-forming toxins, membrane phospholipid damaging toxins, and other cytotoxins and toxins interacting with the immune system involved in gangrene lesion generation.

In recent decades, important progress has been made in the understanding of the molecular and cellular mechanisms of action of bacterial toxins as well as in the genetic approach. More recently, the rapid development of the genetic methodology including whole genome sequencing with high-throughput sequencing techniques, increasing development of biological data banks, computational genome analysis, and appropriate genomic data-mining has allowed a better understanding of the diversity and distribution of toxins through the bacterial world. Thus, a wide epidemiological study based on genotyping of 26 virulence factors in a large number of *Staphylococcus aureus* strains from bovine mastitis improved the knowledge of the diversity of this pathogen. *S. aureus* is a major cause of intramammary infection in dairy cows worldwide. Novel genotypes have been identified, and the geographical distribution of the variant strains was investigated. This study brings new insights in the evolution and dissemination of this pathogen [1]. The diversity and the mechanisms of action of *S. aureus* toxins have been explored in the review of Oliveira et al. [2]. *S. aureus* toxins include numerous pore-forming toxins, specific proteases which destabilize the keratinocyte junctions (exfoliative toxins), and toxins modulating the immune system (superantigens). The role of *S. aureus* and its toxins in atopic dermatitis and eye infections are discussed in [3,4]. *S. aureus* produces a set of superantigen enterotoxins which are responsible for foodborne outbreaks. A recent epidemiological study shows the diversity of the enterotoxin gene profiles of *S. aureus* in a province of China [5]. The knowledge of *S. aureus* toxins is the basis of a better understanding of the epidemiology and pathophysiology of this pathogen, and allows the development of efficient innovative therapeutics.

BoNTs are the most potent poisonous substances. Until recently, BoNTs were considered to be produced only by bacteria from the *Clostridium* genus. Exploring the increasing number of bacterial whole genome sequences with improved bioinformatics approaches allowed the identification of novel BoNT types and BoNT gene sequences in non-clostridial bacteria. The expanding diversity of BoNTs and dissemination of BoNT gene sequences in non-clostridial species is summarized in the review of Tehran and Pirazzini [6]. Albeit being the most dangerous substances, native BoNTs are efficient therapeutic drugs which are used in an increasing number of medical indications. Due to their high potency, precise titration of therapeutic BoNTs is highly critical. The gold standard is still the mouse bioassay, but promising alternative methods are in development [7]. BoNTs act at the neuromuscular junctions by blocking the release of acetylcholine and thus induce flaccid paralysis of the skeletal muscles. Therefore, BoNTs have been initially used in the treatment of dystonias such as localized muscle spasticity. Then, the beneficial effects of therapeutic BoNTs have been expanded to a broad variety of neurological and muscular disorders targeting other cholinergic neurons than motor neurons. For example, BoNTs are indicated in hyperactivity of smooth muscle (detrusor overactivity, bladder overactivity) as well as of exocrine gland (sialorrhea, hyperhidrosis). More recently, BoNTs have been found to be effective on sensory neurons and are used in the treatment of peripheral and central neuropathic pain. The review of Fonfria et al. [8] highlights the available formulations of BoNTs and their expanded medical indications. The diffusion of BoNTs in the locally injected muscle and dissemination to the central nervous system is discussed. The therapeutic effects of BoNT/A on sialorrhea has been further investigated by Restivo et al. [9]. It is shown that the reduction in drooling depends on the number of treated glands. Injections in higher numbers of glands result in more significant sialorrhea reduction. The mechanism of action of the antinociceptive effects of BoNTs is still unclear. The review of Park et al. and Matak et al. [10,11] summarizes the current concepts of the mechanism of antinociceptive effects of BoNTs and reports the clinical studies of therapeutic use of BoNT/A in pain. The effects of BoNTs on pain relief and also on muscle weakening could also result from BoNT activity on the central nervous system as reviewed in [12]. One limit of the therapeutic use of BoNTs is the generation of neutralizing antibodies which can inhibit or reduce the effects of these toxins. This is discussed in [13]. 

In contrast to BoNTs, which inhibit the exocytosis of neurotransmitters by interfering with SNAREs (soluble n-ethylmaleimide-sensitive factor attachment protein receptors), mycolactone, a lipid-like toxin produced by *Mycobacterium ulcerans*, impairs exocytosis in platelets and mast cells by interacting with SNARE regulators. Mycolactone is responsible for the lesions of Buruli ulcer caused by *M. ulcerans,* which is characterized by the absence of wound healing. It is considered that the main mechanism of action of mycolactone is to block the release of platelets and mast cell factors that facilitate wound healing. Using molecular docking and dynamics simulations, Kwofie et al. [14] show that among the SNARE proteins, Munc18b is the preferred target of mycolactone. Thus, they hypothesized that mycolactone prevents exocytosis by inhibition of Munc18b, which is a chaperone protein of syntaxin, a key SNARE of the exocytosis process. Experimental data are expected to support the predicted results.

*Clostridium perfringens* is the pathogen which produces the most numerous toxins and is involved in multiple diseases in man and animals such as gangrene, enteritis, enterotoxemia, septicemia, and food-borne intoxication. In recent years, significant progress has been made in the knowledge of these toxins. The molecular mechanisms of action of cell death mediated by *C. perfringens* toxins are reviewed in the relevant article of Navarro et al. [15].

A specific group of bacterial toxins, called cyclomodulins, interfere with DNA inducing DNA breaks and cell senescence or apoptosis. Among them, colibactin is mainly produced by the B2 phylogroup of *Escherichia coli* strains, which have been associated with extra-intestinal infections (septicemia, newborn meningitis, urinary tract infections). In addition, colibactin is strongly suspected to play a role in colorectal cancer. The recent advances on the genetics, biosynthesis, structure, and mode of action of colibactin are reviewed in the article of [16]. It is intriguing how colibactin, which induces cell cycle arrest, can lead to cancer cell proliferation. It is hypothesized that colibactin induces senescent cells which are metabolically active, and which stimulate proliferation of uninfected cells through increased production of hepatocyte growth factor.

*Helicobacter pylori* is another pathogen responsible for cancer, notably gastric cancer. The oncogenic cytotoxin-associated gene A (CagA) protein is considered as the major toxin involved in *H. pylori* gastric cancer. Epidemiological studies reviewed in [17] show that CagA seropositvity, which reflects *H. pylori* infection, is associated with increased risk of gastric cancer. It is proposed that in regions of high incidence of gastric cancer, screening and treatment of *H. pylori* infection would be feasible and effective to reduce the incidence of this disease.

In addition to medical applications, bacterial toxins are also useful and efficient tools in plant technology. Thereby, bacterial toxins represent a safer and more ecological alternative to chemical insecticides in agricultural practices. Indeed, bacterial toxins such as *Bacillus thuringiensis* insecticidal toxins are specific of insects and do not affect vertebrates, albeit limited toxicity has been observed in certain mammalian cell lines. *B. thuringiensis* synthesize various insecticidal toxins including crystal (Cry) toxins during the sporulation process and vegetative insecticidal proteins (Vip) during the growth phase (see the recent Special Issues "*Bacillus thuringiensis* Toxins: Functional Characterization and Mechanism of Action, 2019"; "Insecticidal Toxins from *Bacillus thuringiensis*, 2019"). Since several decades ago, *B. thuringiensis* is used as a biopesticide in agriculture by spreading a mixture of spores and *B. thuringiensis* toxins. More recently, transgenic crop expressing *B. thuringiensis* toxins, notably Cry, has been developed. However, the wide use of *B. thuringiensis* might lead to the emergence of insect resistance. One strategy to delay insect resistance is to generate transgenic plants expressing several different toxins such as a combination of Cry and Vip proteins. However, Yang et al. showed insect resistance in *B. thuringiensis* transgenic maize expressing both Cry1 and Cry2 and also Cry and Vip3A proteins [18]. Thus, the management of insect resistance is challenging, and better knowledge of the mechanism of resistance is crucial for the development of efficient strategies. A key aspect is the interaction of toxins with insect cells which is controlled by specific recognition of cell surface receptors. As reviewed by Sato et al. [19], ATP-binding cassette (ABC) transporters are considered as receptors of Cry toxins in insects, and mutations in ABC transporters are a major cause of insect resistance to Cry toxins.

The top articles of the two last years highlight the powerful approach of the molecular and bioinformatics methodologies in deciphering toxin variants and their distribution as well as in refining their mechanism of action. Molecular modeling facilitates the understanding of toxin interactions with their receptors and/or targets, notably when these compounds are membrane associated and their biochemical approaches are challenging. The improved knowledge of bacterial toxins impacts the development of efficient countermeasures such as vaccines and specific inhibitors. An increasing aspect of bacterial toxins is their medical and environmental applications, notably, BoNTs which are the most powerful toxins, are among the therapeutic drugs with the largest number of medical indications, as pointed out by the abundant publications on this subject (8 of 19 top articles in this review). This implies that BoNTs have a more complex mechanism of action than the basic blockade of acetyl-choline release at the neuro-muscular junctions. It is more and more apparent that bacterial toxins have not a unique mode of action but exert their toxicity through multiple cellular effects, which in large part remain to be determined. Thus, BoNT activity on sensory neurons and in the central nervous system is still poorly understood. In agriculture, *B. thuringiensis,* which produces a vast range of insecticidal toxins, is increasingly used as a substitute of chemical pesticides. However, the development of insect resistance leads to adapt efficient strategies such as those based on combinations of several *B. thuringiensis* toxins and also requires a better understanding of *B. thuringiensis* toxin interaction with target cells. In addition, certain *B. thuringiensis* toxins are cytotoxic for human cancer cell lines and could be novel anticancer agents (reviewed in [20]). Thereby, bacterial toxins represent a promising source of tools as native toxins or engineered compounds for various applications.
